# Mulberry Leaf Reduces Oxidation and C-Reactive Protein Level in Patients with Mild Dyslipidemia

**DOI:** 10.1155/2013/787981

**Published:** 2013-01-15

**Authors:** Pornanong Aramwit, Ouppatham Supasyndh, Tippawan Siritienthong, Nipaporn Bang

**Affiliations:** ^1^Bioactive Resources for Innovative Clinical Applications Research Unit, and Department of Pharmacy Practice, Faculty of Pharmaceutical Sciences, Chulalongkorn University, PhayaThai Road, Phatumwan, Bangkok 10330, Thailand; ^2^Division of Nephrology, Department of Medicine, Phramongkutklao Hospital and College of Medicine, Bangkok 10400, Thailand

## Abstract

C-reactive protein (CRP) is the inflammatory marker that could represent the inflammation in blood vessels resulted from dyslipidemia. The objective of this study was to evaluate the antioxidative activity of mulberry leaf powder using DPPH assay and the effect of mulberry leaf powder on lipid profile, CRP level, and antioxidative parameters in mild dyslipidemia patients. A within-subjects design was conducted and patients received three tablets of 280 mg mulberry leaf powder three times a day before meals for 12 weeks. Total of 25 patients were enrolled but one subject was excluded. After three months of mulberry leaf consumption, serum triglyceride and low-density lipoprotein (LDL) level were significantly reduced and more than half of all patients' CRP levels decreased every month as well as the mean CRP level but no statistically significant difference was found. The average erythrocyte glutathione peroxidase activity of patients was increased but not at significant level; however, the mean serum 8-isoprostane level was significantly lower after mulberry treatment for 12 weeks. It can be concluded that mulberry leaf powder exhibited antioxidant activity and mulberry leaf powder has potential to decrease serum triglyceride, LDL, and CRP levels in mild dyslipidemia patients without causing severe adverse reactions.

## 1. Introduction

Mulberries (*Morus alba* L., Moraceae) have been widely used in traditional Oriental medicine for several applications including prevention of diabetes [1]. It contains various nutritional components such as flavonoids and polyphenols, especially 1-deoxynojirimycin (DNJ), a potent glucosidase inhibitor, which shows hypoglycemic [2], hypolipidemic [3], and antiatherogenic effects [4, 5] in certain animal models. Shibata et al. [6] reported that mulberry leaf-derived aqueous fractions inhibit tumor necrosis factor-*α*-induced nuclear factor *κ*B activation and lectin-like oxidized low-density lipoprotein receptor-1 expression in vascular endothelial cells. Harauma et al. [5] also found that mulberry leaf powder can prevent atherosclerosis in apolipoprotein E-deficient mice. Our earlier research also found that mulberry leaf powder was effective in reducing lipid profile in mild hyperlipidemia patients [3]. Even though mulberry leaf powder seems to have several advantages in cardiovascular diseases, very few studies have been investigated in human subjects. 

Dyslipidemia with high serum cholesterol, both total cholesterol and low-density lipoprotein (LDL), and low high-density lipoprotein (HDL), is a critical cardiovascular risk factor [7, 8]. It is now established that oxidation of LDL constitutes a key event in inflammation and atherogenesis [9]. Mechanisms of LDL oxidation involve concerted modification by oxidants produced by arterial wall cells, such as reactive nitrogen species, hydroxyl radicals, and lipid-soluble free radicals [10]. Because most mechanisms involve the oxidation process, antioxidants may be useful in preventing endothelium blood vessel related to atherosclerosis. 

There is extensive evidence that link hypercholesterolemia with increased lipid peroxidation and increased oxidative stress [11, 12]. The oxidative modification of lipoproteins, particularly LDL, has emerged as a fundamental process in the development of atherosclerosis [13]. Oxidative stress due to increased reactive oxygen species (ROS) generation and unbalanced oxidative/antioxidative equilibrium are also implicated in the development of coronary arteriosclerotic cardiovascular disease especially in patients with hyperlipidemia [14]. Glutathione peroxidase (Gpx) is considered as one of the primary defense systems which eliminate excess ROS and maintain equilibrium between oxidative and antioxidative activity under normal physiological conditions [15] while 8-isoprostane, the final esterified product of oxidized arachidonic acid, seems to be a good marker of oxidative injury and is considered a gold standard for measuring oxidative stress *in vivo*.

C-reactive protein (CRP), generated from the liver, is a protein in the kind of acute phase reactant. It will respond immediately after the rising of nonspecific inflammation in the blood vessel [16, 17]. CRP has been identified in human atherosclerotic lesions and has been hypothesized to mediate endothelial dysfunction through induction of endothelial adhesion molecules, tissue factor, and proinflammatory cytokine synthesis [18]. Accordingly, CRP in blood can reflect the process involved with inflammation and it is a strong independent predictor of future peripheral arterial disease [19], myocardial infarction, and stroke among apparently healthy men and women [20]. As a marker of inflammation, CRP measurement has been recommended in cardiovascular risk stratification and clinical treatment guidelines, additional to traditional cardiovascular risk assessment [21, 22]. When combined with lipid screening, CRP improves global risk prediction in patients who would not be identified for primary prevention of cardiovascular events by lipid assessment alone [23]. 

The purposes of this study are to evaluate the antioxidant property of mulberry leaf-derived aqueous fraction *in vitro*; the effect of mulberry leaf on the serum lipid profile, erythrocyte glutathione peroxidase activity, and 8-isoprostane, and the effect of mulberry leaf in reducing the CRP levels in patients with mild dyslipidemia.

## 2. Materials and Methods

### 2.1. Materials

Mulberry leaf powder and tablets were derived from Kitayamakit Co. Ltd. (Kyoto, Japan). They were produced from chemical-free mulberry leaves. Each tablet weighed 280 mg and contained 254.8 mg mulberry leaf powder, which had 0.367 mg of the active ingredient DNJ.

### 2.2. Methods

#### 2.2.1. Analysis of Antioxidant Activity

Antioxidant activity of mulberry leaf was determined by free radical scavenging activity using the 1,1-diphenyl-2-picrylhydrazyl (DPPH) assay [24]. Mulberry leaf powder (0.3 g) was extracted in 3 mL of hot water (95°C) with continuous shaking for 30 minutes. After getting cool at room temperature, it was centrifuged at 5,000 rpm for 20 minutes then filtered through 0.45 *μ*m filter. The supernatant obtained was considered as undiluted mulberry leaf extract (100%). Serial dilution of undiluted extract was performed in order to obtain extract at 50, 25, 12.5, and 6.25%, respectively. Then 100 *μ*L of different concentrations of mulberry leaf extracts were mixed with 150 *μ*L of 0.1 mM DPPH methanolic solution. The samples were shaken vigorously and allowed to stand for 20 minutes at room temperature. A control was prepared without any sample and purified water was used for baseline correction. The decrease in the absorbance of the formed blue to violet reagent was determined after 20 minutes at 550 nm and the percentage inhibition activity was calculated from the following equation:

(1) $$\begin{array}{c} \;\left(\frac{\left(A_{0}-A_{1}\right)}{A_{0}}\right)\;\times \;100, \end{array}$$

where *A*_0_ = Absorbance of the control and *A*_1_ = Absorbance of the extract/standard.

 Experiments were carried out 5 times. Data were expressed as the half maximal inhibitory concentration (IC_50_), which is the concentration of an antioxidant at which 50% inhibition of free radical activity is observed. 

#### 2.2.2. Subjects and Study Design

A within-subjects research design was conducted at the outpatient internal medicine clinic, Phramongkutklao Hospital, Thailand. All patients were screened at the beginning of the study. Eligibility criteria for entry into the study included the following: (1) age between 20 and 60, (2) met the National Cholesterol Education Program Adult Treatment Panel III (NCEP ATP III) criteria for dyslipidemia [9, 25], (3) must have serum LDL level in the range of ≥ 140 and < 190 mg/dL and fasting plasma glucose < 126 mg/dL, (4) should not have more than one major cardiovascular disease (CVD) risk factor according to NCEP ATP III guidelines, (5) must not be receiving lipid lowering drugs except for diet control, only diuretics were allowed for patients with hypertension and their blood pressure had to be controlled at level < 140/90 mmHg, and (6) should have liver enzymes (alanine aminotransferase and aspartate aminotransferase) < 40 U/L and blood urea nitrogen < 20 mg/dL, with serum creatinine in the range of 0.6–1.2 mg/dL. The patients with the following criteria were excluded from the study: (1) patients needing to receive lipid lowering drugs according to NCEP ATP III guidelines and if they had a high risk factor for CVD or equivalent, (2) had a Framingham risk score greater than 20%, (3) had abnormal liver or renal function tests, (4) had severe complications or had been admitted to the hospital for cardiovascular events in the three months prior to enrollment in this study, and (5) patients with cancer and those who were pregnant or breastfeeding. Written informed consent was obtained from all study participants after a thorough discussion of the protocol, its rationale, and potential risks. The protocol was approved by the Ethics Committee of the Institutional Review Board of Phramongkutklao Hospital.

Prior to the enrollment, all subjects were asked about underlying diseases, current medications, and personal profile. On the first visit of the screening period, blood samples were collected from all patients and a dietician advised them during the four-week period of diet control. This advice included the diet consumed, food exchange, and a diet that was appropriate for each patient, and they were carefully instructed on how to record their total oral intake using household measures. Each patient was requested to make a three-day food record by recording all of the food and beverages that they consumed over two working days and one day of the weekend. Blood samples were collected to determine the lipid profile after four weeks of diet control. Patients who reached the target lipid profile after diet control were withdrawn from the study while patients who could not reach the target according to NCEP ATP III guidelines continued in this study and the lipid profile before receiving mulberry leaf tablet therapy was examined. All included subjects were assigned to receive three tablets of 280 mg mulberry leaf powder three times a day before meals, the dose which has been proven to reduce the LDL in mild dyslipidemia patients [3], for 12 weeks. CRP measured by high-sensitivity methods was used in order to measure low levels of CRP more accurately. Briefly, blood samples were completely coagulated and centrifuged as serum and then stored frozen at −80°C. The CRP levels were determined by particle-enhanced immunonephelometry. The serum sample of 200 *μ*L was diluted and reacted with CRP reagent (suspension of polystyrene particles coated with mouse monoclonal antibodies to CRP) to form an immune complex. A beam of light at the wavelength of 840 nm was then passed through the sample; the amount of light scattering was measured by photodetector. The intensity of the scattered light was proportional to the concentration of the relevant protein in the samples. The result was evaluated by comparing with a standard of known concentration. Routine blood analyses including lipid parameters, CRP, fasting plasma glucose, and liver function tests of all subjects were performed every four weeks. Clinical evaluation for side effects and pill counts to determine compliance were also performed every four weeks at the follow-up visits. If total pill count indicated more than 20% of mulberry leaf tablets untaken, subjects were excluded from the study. 

Glutathione peroxidases (Gpx) was measured by the modified method of Paglia and Valentine [26] as described by Jacobson et al. [27]. The rate of glutathione oxidation was measured by monitoring the disappearance of NADPH+H^+^ in the reaction medium (decrease of absorbance at 340 nm), since NADPH+H^+^ was consumed for the reduction of oxidized glutathione by glutathione reductase. 8-isoprostane concentrations were measured in duplicate using a specific enzyme immunoassay kit (Cayman Chemicals, Ann Arbor, MI, USA). The detection limit was 5 pg/mL and the intraassay and interassay variabilities were 6 and 7%, respectively.

#### 2.2.3. Statistical Analysis

Results were expressed as mean and standard deviations. The *t*-test was used to evaluate the difference between pre- and posttreatment. Repeat-measures one-way ANOVA by Bonferroni was used to test the differences in CRP levels in each period. A *P* < 0.05 was considered statistically significant.

## 3. Results

**Figure 1:**
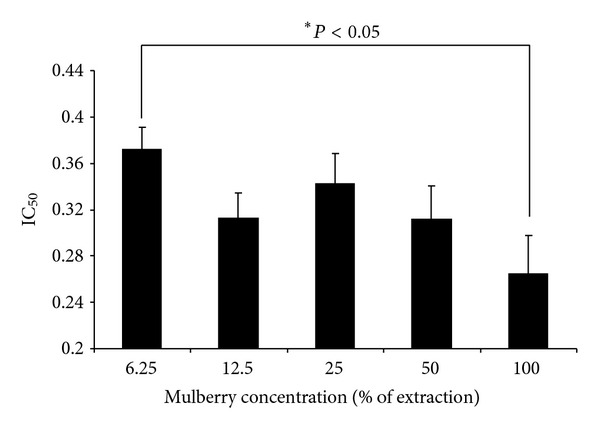
IC_50_ of mulberry leaf extracts at room temperature.

Free radical scavenging activities at room temperature from mulberry leaf extracts, indicated as IC_50_, are shown in Figure 1. The results showed that mulberry leaf extracted by hot water exhibits good antioxidant activity. Undiluted mulberry leaf extracts exhibit the highest free radical scavenging activities (the lowest IC_50_ value) while the most diluted mulberry leaf extracts show the lowest free radical scavenging activities. The concentration of mulberry leaf extracts correlates well with their antioxidant activities. There is a significant difference in IC_50_ of 100% and 6.25% mulberry leaf extract.

Twenty-five subjects have enrolled in the study.  Table 1 summarizes the demographic characteristics and baseline laboratory data of all participants in this study. None of the participants had underlying disease or current medications. The mean age was 35.88 years with the majority being female. Generally, it can be concluded that the subjects with mild dyslipidemia and having normal body weight were at low risk of coronary heart disease.

Most subjects complied with their medication regimens. The percentage of compliance ranged from 89.63 to 98.52% with the mean of 95.00%. One subject was eliminated from the study since she discontinued her lifestyle modification by consuming a high fat diet routinely after starting the mulberry leaf tablet regimen. Thus, her lipid level could increase and affect the CRP level to be higher than usual.

Table 2 represents the serum lipid profile in patients with mild dyslipidemia after 12 weeks of treatment with mulberry leaf. After 12 weeks of treatment, serum triglyceride and LDL level were significantly reduced by approximately 10.6% and 8.2%, respectively (*P* < 0.05), from baseline, while diet control did not improve lipid profile. Moreover, the HDL was increased by 6.3% even though no significant difference was found. 

**Figure 2:**
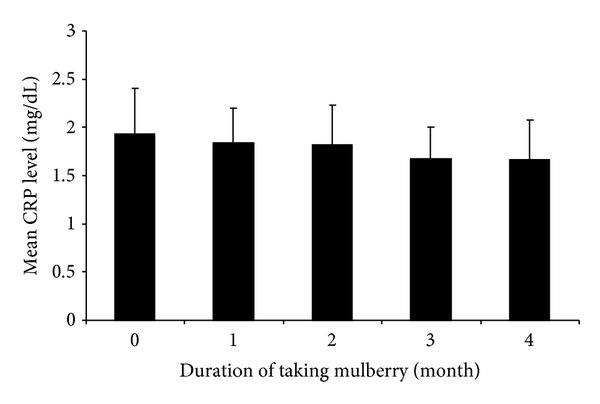
Mean CRP level of subjects in each month (*n* = 24).

According to the monthly CRP blood level, more than half of all patients' CRP levels decreased every month compared to the CRP level before the intake of mulberry leaf tablets as indicated in Table 3. The mean CRP level of subjects also decreased every month as shown in Figure 2. After three months of mulberry leaf tablet consumption, 16 patients (66.67%) had lower CRP levels compared to baseline after lifestyle modification. However, there is no statistically significant difference of CRP level between each month. 

The average erythrocyte glutathione peroxidase activity of patients at baseline (after diet control) was 12.16 ± 3.54 U/g Hb while this parameter increased to 14.22 ± 2.86 U/g Hb after mulberry treatment for 12 weeks but no significant difference was found. The level of 8-isoprostane showed the same trend; however, the significance difference between baseline and after mulberry treatment was found. At baseline the average 8-isoprostane level was 563.12 ± 14.16 pg/mL and the value decreased to 244.68 ± 22.17 pg/mL after mulberry treatment. 

Twelve adverse reactions from mulberry leaf tablet consumption were observed. Details are shown in Table 4. Most common adverse reaction was diarrhea, which could occur on the first day of the mulberry consumption. However, the diarrhea, as well as other symptoms, was considered as minor and the patients were able to well tolerate mulberry leaf consumption after one week. All conditions disappeared after the patients followed the advice by taking mulberry leaf tablets immediately after meals. No severe adverse reaction was found in this study.

## 4. Discussion

Our present study indicated that mulberry leaf tablet therapy is more effective than diet control alone for controlling lipid profile in mild dyslipidemia patients as shown by a significant fall in serum triglycerides and LDL as well as total cholesterol/HDL ratio. It also showed a rise in HDL in all patients and it is well known that improving the lipid profile potentially reduces the risk of major cardiovascular events.

C-reactive protein can be used as a marker of acute inflammation and it also has been widely used for monitoring disease activity in cardiovascular disease and diabetes [28–30], which emphasizes the likely role of chronic inflammation in the aetiology. C-reactive protein also rises with vascular insufficiency and damage of most types, which includes acute myocardial injury or infarction, stroke, and peripheral vascular compromise. Elevation of the CRP level has predictive value for an increased risk of an acute coronary event compared to very low CRP levels [31]. Regarding to gender, women normally have significantly higher CRP levels than men [32] and stronger correlation was found in women between the association of obesity and CRP level [33]. Our results indicated that mulberry powder extracted exhibited strong antioxidant property, which is similar to results found by others [5, 34]. Even though earlier report showed that mulberry leaf extracted by water had very little effect on cell cycle progression and had less 2,2-diphenyl-1-picrylhydrazyl radical scavenging activities compared to mulberry leaf extracted by methanol or butanol [35], it still shows strong free radical scavenging activity *in vitro*. From our result, it could be stated that lifestyle modification can prevent more inflammation in blood vessel that occurs from dyslipidemia resulting in the decreased CRP levels especially when the intake of the mulberry leaf tablet was included. We found that CRP mean level in subjects receiving mulberry leaf tablets had a tendency to decrease every month, especially after 12 weeks of mulberry leaf consumption. It could be stated that mulberry leaf tablet consumption can prevent more inflammation of blood vessels that occurs from dyslipidemia. However, the difference of CRP levels in each month was not statistically significant which may be due to a small number of the participants and the initial CRP levels in all subjects were rather low. Even though most of subjects in this study were female who has high tendency of elevated CRP, most subjects were considered as normal (healthy weight) according to their body mass index resulting in small-elevated CRP. Despite its high sensitivity, it has only 19-hour half-life which might have caused deviation [36]. However, according to patient interviews, there was no illness, inflammation or infection, injury, or medication use during the study period, which could relate to their particular CRP levels.

Maximum CRP levels > 3 mg/dL had positive predictive values > 20% for proven or probable early-onset infections or inflammation [37]. Only 5 out of 25 patients in our study had CRP levels higher than 3 mg/dL at initial stage with no acute phase reaction of inflammation or infection reported in their patient profiles and their CRP levels after 3 months of mulberry consuming reduced significantly in all patients and the levels are within normal range (data not shown). In month 3, the average CRP levels decreased only slightly, possibly due to the fact that most subjects had mild dyslipidemia with low risk of coronary heart disease and their initial CRP levels were rather low. The intake of mulberry leaf tablet could, thereby, reduce CRP levels to a certain extent. 

Taking other factors into consideration, one patient who took oral contraceptives during the research participation exhibited a high initial CRP level. This might be due to the estrogen/progestogen hormone use. After changing her lifestyle and consuming mulberry leaf tablets, her CRP level continued to decrease and reduced by 77% in the third month (data not shown). This might, therefore, be an indication that the decrease in CRP level will be obvious when the patient's initial CRP level was high.

Glutathione peroxidase activity is a key antioxidant enzyme within most cells including endothelial cells. Due to its important role in the prevention of oxidative stress, it is considered to be an antiatherogenic enzyme [38]. Guo et al. [39] showed that reduced expression of Gpx resulted in an increase of cell-mediated oxidation of LDL in mice. In human, the lack of Gpx activity in atherosclerotic lesions appeared to be associated with the development of more severe lesions [40]. Our results indicate that mulberry leaf enhances the antioxidant activity in human by increasing Gpx activity even though no significant difference between before and after mulberry consumption was found which may possibly due to; again, most subjects had only mild dyslipidemia with no severe life-threatening condition.

The isoprostanes are a unique series of prostaglandin-like compounds formed *in vivo* via a nonenzymatic mechanism involving the free radical-initiated peroxidation of arachidonic acid [41]. 8-isoprostane has been focused due to its stability, specificity for lipid peroxidation, and relative abundance in biological fluid [42]. In this present study, the mean serum 8-isoprostane level was significant lower after mulberry treatment for 12 weeks despite that its initial serum level at the baseline was much higher. This observation may verify the notion that mulberry leaves possess antioxidative properties in clinical application. 

During this study, there was no severe adverse reaction found. A minor diarrhea occurred, however, possibly due to the fact that mulberry leaf tablets are rich in dietary fiber, which could stimulate defecation. Accordingly, constipation is also possible when there is insufficient water in the bowels' contents. An active ingredient in mulberry leaf, DNJ, also acts as alpha-glucosidase inhibitor, which prevents disaccharide digestion, thereby causing gastrointestinal side effects such as flatulence or abdominal distention. Moreover, mulberry-induced reduction in blood glucose might increase appetite or cause dizziness.

## 5. Conclusions

Extraction of mulberry leaf powder by hot water exhibited strong antioxidative activity. This research also reveals the tendency of mulberry leaf powder in reducing serum LDL and triglyceride as well as blood vessel inflammation stemmed from dyslipidemia, by the measurement of decreased CRP levels. Moreover, mulberry leaf powder can increase the erythrocyte glutathione peroxidase activity and decrease 8-isoprostane in serum. No severe adverse reaction was found and minor side effects can be relieved by taking mulberry leaf tablets immediately after meals.

## Figures and Tables

**Table 1:** Patient demographic characteristics and baseline laboratory data (*n* = 25).

Characteristics	
Gender (M : F)	5 : 20
Age (years)	35.88 ± 10.87
Body mass index (kg/m^2^)	23.18 ± 3.12
Fasting plasma glucose (mg/dL)	92.14 ± 6.72
Alanine aminotransferase (U/L)	18.42 ± 3.25
Aspartate aminotransferase (U/L)	17.44 ± 3.62
Serum creatinine (mg/dL)	0.62 ± 0.04
Blood urea nitrogen (mg/dL)	11.79 ± 2.73
CVD risk factor (none : 1 risk)	21 : 5

**Table 2:** Serum lipid profile in patients with mild dyslipidemia after 12 weeks of treatment (*n* = 24).

	Mulberry leaf tablet therapy		
	Baseline	Week 12	Change (%)
Total cholesterol (mg/dL)	216.3 ± 6.1	211.5 ± 4.3	−1.8
Triglyceride (mg/dL)	114.2 ± 8.7	86.2 ± 7.4	−10.6*
Low-density lipoprotein (mg/dL)	163.7 ± 5.1	151.2 ± 3.8	−8.2*
High-density lipoprotein (mg/dL)	41.5 ± 3.4	45.3 ± 1.9	+6.3
TC : HDL	5.6 ± 0.2	3.1 ± 0.3	−6.4*

TC: total cholesterol, HDL: high-density lipoprotein.

*P < 0.05 compared with the baseline.

**Table 3:** Mean difference of CRP level and number of patients whose CRP level decreased comparing to baseline (month 0, after diet therapy) (*n* = 24).

Duration	Mean difference of CRP level (mg/L) ± SD	Number of patients with decreased CRP (%)
Month 1–month 0	−0.0175 ± 0.898	14 (58.33)
Month 2–month 0	−0.1606 ± 1.201	14 (58.33)
Month 3–month 0	−0.1753 ± 1.180	16 (66.67)

**Table 4:** Incidence of adverse reactions from the mulberry leaf powder treatment (*n* = 25).

Adverse reactions	Number of patients (%)
Gastrointestinal	
Flatulence	1 (4)
Diarrhea	6 (24)
Constipation	1 (4)
Miscellaneous	
Increase appetite	2 (8)
Dizziness	2 (8)
